# Human immunodeficiency virus type 1 efficiently binds to human fetal astrocytes and induces neuroinflammatory responses independent of infection

**DOI:** 10.1186/1471-2202-8-31

**Published:** 2007-05-12

**Authors:** Jinliang Li, Galina Bentsman, Mary Jane Potash, David J Volsky

**Affiliations:** 1 Molecular Virology Division, St. Luke's-Roosevelt Hospital Center and Columbia University, 432 West 58^th ^Street, New York, NY 10019, USA; 2 Beckman Research Institute of the City of Hope, Division of Virology, 1500 East Duarte Road, Duarte, CA 91010-3011, USA

## Abstract

**Background:**

HIV-1 infects human astrocytes *in vitro *and *in vivo *but the frequency of infected cells is low and its biological significance is unknown. In studies *in vitro*, recombinant gp120 alone can induce profound effects on astrocyte biology, suggesting that HIV-1 interaction with astrocytes and its functional consequences extend beyond the limited levels of infection in these cells. Here we determined the relative efficiencies of HIV-1 binding and infection in human fetal astrocytes (HFA), mainly at the single cell level, using HIV-1 tagged with green fluorescence protein (GFP)-Vpr fusion proteins, termed HIV-GFP, to detect virus binding and HIV-1 expressing Rev and NefGFP fusion proteins to detect productive infection.

**Results:**

Essentially all HFA in a population bound HIV-GFP specifically and independently of CCR5 and CXCR4. The dynamics of this binding at 37°C resembled binding of an HIV fusion mutant to CD4-positive cells, indicating that most of HIV-GFP arrested infection of HFA at the stage of virus-cell fusion. Despite extensive binding, only about 1% of HFA were detectably infected by HIV-RevGFP or HIV-NefGFP, but this proportion increased to the majority of HFA when the viruses were pseudotyped with vesicular stomatitis virus envelope glycoprotein G, confirming that HFA impose a restriction upon HIV-1 entry. Exposure of HFA to HIV-1 through its native proteins rapidly induced synthesis of interleukin-6 and interleukin-8 with increased mRNA detected within 3 h and increased protein detected within 18 h of exposure.

**Conclusion:**

Our results indicate that HIV-1 binding to human astrocytes, although extensive, is not generally followed by virus entry and replication. Astrocytes respond to HIV-1 binding by rapidly increased cytokine production suggesting a role of this virus-brain cell interaction in HIV-1 neuropathogenesis.

## Background

Human immunodeficiency virus type 1 (HIV-1) infection is associated with a spectrum of neurological diseases of varying severity including the endstage syndrome HIV-associated dementia (HAD) [1]. Although the core pathophysiological defects of HAD are neuronal damage and loss of specific neuronal populations [1-4], neurons rarely show evidence of HIV-1 infection [5-7]. It is generally accepted that HIV-1 can be neuropathogenic through synthesis of viral proteins that are directly neurotoxic as well as through an array of cellular toxins that are produced by HIV-1-infected cells (reviewed in [8,9]). Particularly in the case of HAD with encephalitis, it is clear that HIV-1-infected macrophages contribute greatly to disease (reviewed in [8,10,11]). In various model systems and in HIV-1-infected humans, multiple products of macrophages have been associated with neuropathogenesis including arachidonic acid metabolites, IL-6, MCP-1, platelet activating factor, and TNF-α [12-16].

There is growing interest in the potential role of astrocytes in HIV-1-mediated neuropathogenesis. Astrocytes are an abundant [17] and heterogeneous [18,19] population of cells of neuroectodermal origin which perform many essential functions in the brain, from structural and metabolic support, responses to brain injury and innate immune reactions, control of extracellular glutamate, to regulation of neuronal cell activities and neural signaling (reviewed in [20-23]). Astrogliosis, the presence of activated and hypertrophied astrocytes, is a defining neuropathological characteristic of HAD [24,25]. There is also evidence of HIV-1 infection in a small and variable fraction of astrocytes *in vivo*, particularly in advanced brain disease [7,26-30]. The significance of these overt astroglial pathologies is unknown but overall, unlike neurons, astrocytes rarely die in HIV-1-infected brains [31,32]. Productive infection of human astrocytes with HIV-1 has significant effects on cell physiology *in vitro *[33,34] and it associates with measurable neuropathology in a mouse model [35], suggesting that infected astrocytes, although infrequent, can have localized pathogenic effects.

Growing evidence suggests that astrocytes also may suffer dysregulation in the HIV-1-infected brain that may extend beyond the limited levels of HIV-1 infection and contribute to neuropathogenesis in distinct pathways (reviewed in [21,36-38]). As part of brain parenchyma, astrocytes are likely exposed continuously to HIV-1 particles, viral proteins, cytokines, and other substances secreted by HIV-1-infected macrophages and microglia. Although they lack CD4 they express CXCR4, and under certain circumstances, CCR3 and CCR5, the co-receptors for HIV-1 entry into cells (reviewed in [39-41]). These chemokine receptors can transduce responses to chemokines and to HIV-1 gp120 present in the brain and they might be involved in HIV-1 association with astrocytes. Studies *in vitro *indicate that many of these products significantly modulate astrocyte physiology which in turn can alter essential interactions of astrocytes with other cells in the brain, particularly neurons. For example, exposure of cultured astrocytes to HIV-1, recombinant gp120, or viral transactivator Tat induces some of the same secretable mediators of neuropathogenesis as those produced by macrophages, including inflammatory cytokines TNF-α and IL-1β, chemokines MCP-1 and IP-10, IL-6, or neurotoxin nitric oxide [42-50]. The apparent dysregulation of astrocyte immune functions could contribute to the overall inflammatory environment in the brain. In other studies in culture, intact HIV-1 or exogenous gp120-induced extensive changes in astrocyte gene expression [38,51-53] and impaired transport of extracellular glutamate by astrocytes [54,55], a defect which may lead to neuronal death by glutamate excitotoxicity [20]. Glutamate uptake can also be impaired by intracellular expression of recombinant Tat or exposure of astrocytes to TNF-α [56,57]. In another potential neurotoxic mechanism, recombinant gp120 was shown to induce Ca^++^-dependent glutamate secretion by astrocytes and neuronal cell death in glial-neuronal co-cultures in a pathway involving signaling through CXCR4 and production of TNF-α [58,59]. Overall, these findings suggest that astrocytes rendered dysfunctional by exposure to HIV-1 in the brain have the capacity to injure or impair neurons. Because both HIV-1 binding and native infection can affect astrocyte function *in vitro *and *in vivo *[33,35,55], astrocytes have a pathogenic potential that exceeds their susceptibility to HIV-1 infection.

The interaction of HIV-1 with astrocytes is somewhat paradoxical. Astrocytes do not express surface CD4 but can bind gp120 with high affinity through an unidentified receptor [60,61]. Although exposure to virus or gp120 can globally alter astrocyte function [53-55], HIV-1 infection is highly inefficient compared to T lymphocytes [62-64], a defect which can be attributed to poor virus entry [65-67]. We performed the present study to directly examine the dichotomy between HIV-1 binding and infection in astrocytes, in part by analysis at the single cell level. We constructed several HIV-1 species that either carry or express GFP fused to viral proteins to evaluate binding and infection of primary HFA by fluorescence microscopy. We also used HIV-1 pseudotyped with the envelope glycoprotein G of vesicular stomatitis virus (VSV-G) to efficiently infect CD4-negative HFA [66] to compare productive infection to virus binding for induction of cellular genes. Our results demonstrate that virtually all HFA bind HIV-1 specifically, remarkably stably, and independently of the chemokine receptors CCR3, CCR5, and CXCR4. As previously reported [68,69], fewer than 1% of HFA were susceptible to HIV-1 infection, but in contrast to some previous studies [70] efficiently expressed Rev-dependent viral RNA and cognate protein. Finally we show that HIV-1 binding to HFA through gp120 is necessary and sufficient to rapidly induce IL-6 and increase expression of IL-8. Our findings provide strong support for a robust pathway of neuropathogenesis following frequent binding of HIV-1 to astrocytes.

## Results

### Specific and extensive binding of HIV-1 by HFA

**Figure 1:**
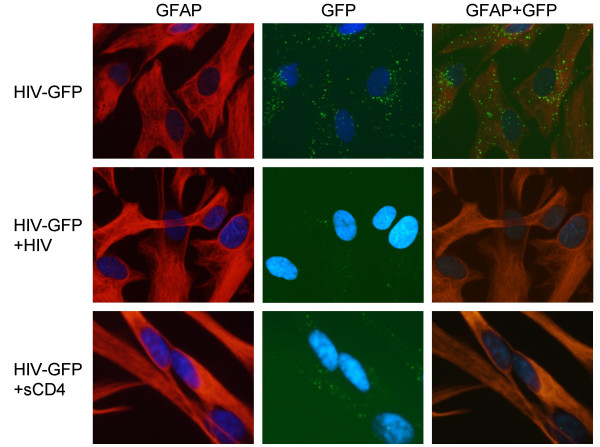
**Extent and specificity of HIV-1 binding to HFA**. Primary HFA were exposed for 1 h at 4°C to NL4-3 carrying GFP-Vpr alone (first row), in the presence of excess unlabeled NL4-3 (second row) or in the presence of soluble CD4 (third row). Cells were stained with anti-GFAP and a Texas Red-labeled secondary antibody to label astrocytes and with DAPI to visualize nuclei. Images of red and green fluorescence were superimposed (third column).

**Figure 2:**
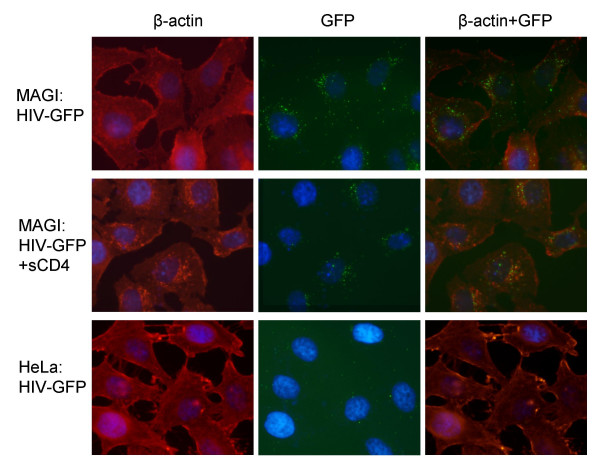
**Extent and specificity of HIV-1 binding to CD4-positive and negative HeLa cells**. CD4-positive MAGI cells were exposed for 1 h at 4°C to NL4-3 carrying GFP-Vpr alone (first row) or in the presence of soluble CD4 (second row). CD4-negative HeLa cells were exposed to HIV-1/NL4-3 carrying GFP-Vpr (third row). Cells were stained with anti-β actin and a Texas Red-labeled secondary antibody and DAPI and images of red and green fluorescence were superimposed (third column).

**Figure 3:**
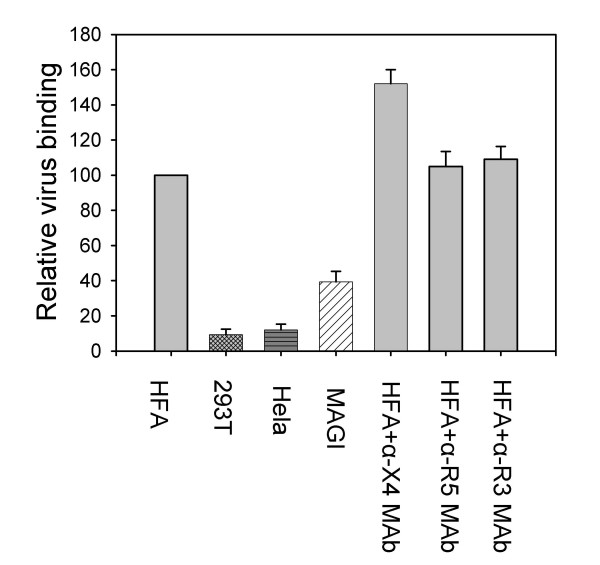
**Quantitation and specificity of HIV-1 binding to HFA**. HFA were exposed for 1 h at 4°C to NL4-3 alone or in the presence of anti-CCR3, anti-CCR5, or anti-CXCR4 antibodies. For comparison, CD4-negative HeLa or 293T cells or CD4-positive MAGI cells were also exposed to NL4-3. HIV-1 binding, as determined by the amount of p24 core antigen associated with cells, was measured by ELISA. Data are expressed relative to HFA.

In previous studies we inferred that HIV-1 interacts with the majority of HFA because exposure to intact or UV-inactivated virus, or recombinant gp120 alone, reduced glutamate uptake by cells and altered cellular gene expression on a population-wide basis [51,71], however such findings are indirect. Using an approach developed by McDonald and colleagues [72], we prepared fluorescently-labeled infectious virions by co-transfection of plasmids encoding intact HIV-1/NL4-3 and a GFP-Vpr fusion protein that is efficiently encapsidated [72] and evaluated this HIV-GFP binding to HFA by fluorescence microscopy. To investigate the specificity of the virus-cell binding, astrocytes were exposed to HIV-GFP in the presence of either unlabeled virus or recombinant soluble CD4 (sCD4) to compete for virion gp120 (Fig. 1). Essentially all HFA bound HIV-1 and this binding was completely blocked by both competitors. To validate our method and reagents, similar studies were carried out with CD4-negative HeLa cells or with MAGI cells, a HeLa derivative engineered to express human CD4 [73] (Fig. 2). HeLa cells failed to bind HIV-1 but MAGI cells bound virus and this inhibition was sensitive to competition by sCD4, indicating that the evaluation of GFP-Vpr-labeled virus binding by microscopy is reliable. These experiments were repeated three times with similar results. We also quantified binding on a population-wide basis by measuring cell-associated p24 1 h after HIV-1 exposure to cells at 4°C (Fig. 3). The extent of binding is expressed relative to the amount of p24 bound to HFA. MAGI cells bound roughly 40% p24 bound to HFA, while CD4-negative 293T or HeLa cells bound less than 15% as much p24 as did HFA. We also exposed HFA to antibodies to the chemokine receptors CCR3, CCR5, or CXCR4, which have been variably detected on HFA [74,75], to test whether any of these receptors are involved in HFA binding of HIV-1. There was a modest increase of virus binding after cells were pretreated with anti-CXCR4; the other antibodies had no effect. Taken together our studies demonstrate that HIV-1 labeled through GFP-Vpr interacts specifically with human cells and that the vast majority of HFA are competent to bind HIV-1. Competitors for gp120 block HIV-1 binding to HFA, confirming the specificity of the interaction of HIV-1 with HFA, however our studies do not identify the cellular receptor.

### HIV-1 internalizes poorly in HFA compared to CD4-positive cells

**Figure 4:**
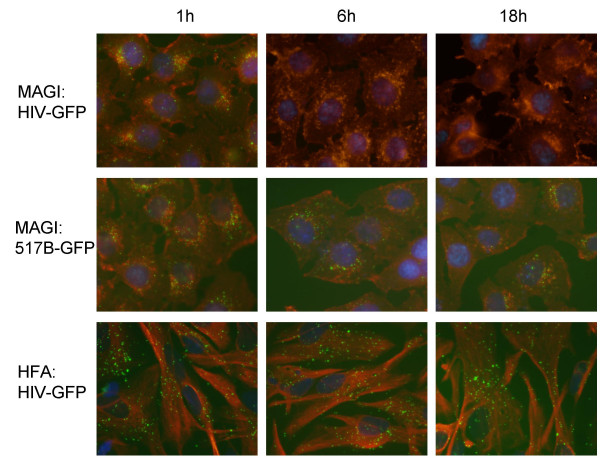
**Dynamics of GFP-Vpr/HIV-1 association with HFA or MAGI cells at 37°C**. MAGI cells were exposed to NL4-3 carrying GFP-Vpr (first row) or to the fusion incompetent HIV-1/517B carrying GFP-Vpr (second row) or HFA were exposed to NL4-3-GFP-Vpr. Cells were incubated with virus at 37°C and fixed at 1 h (first column), 6 h (second column), or 18 h (third column) after infection. HeLa cells were stained with anti-actin and HFA were stained with anti-GFAP, with DAPI and with Texas Red-labeled secondary antibodies. Images shown are superimpositions of red and green fluorescence.

HIV-1 binding to CD4-bearing lymphocytes is rapidly followed by virus-cell fusion and internalization of the viral nucleocapsid [76]. Since it appears that HFA universally bind HIV-1 (Figs. 1, 2, 3) but they permit limited virus infection [63,64], one defect may lie in post-binding events leading to virus entry. To investigate the internalization of HIV-1 by HFA, we exposed HFA or MAGI cells to HIV-GFP in culture at 37°C for various periods of times prior to fixation and observation by fluorescence microscopy (Fig. 4). As a negative control in these studies, we employed 517B-GFP, a mutant of HIV-1 able to bind to but not fuse with cells [77]. HIV-GFP was detected on the surface of both HFA and MAGI cells 1 h after exposure but subsequently the progress of virus-cell interaction in the two cell types diverged. By 6 h after exposure the virus was internalized into MAGI cells and appeared to colocalize or overlay intracellular actin; 18 h after exposure no fluorescent signal was detected in MAGI cells, indicating intracellular breakdown of GFP-Vpr (top panel in Fig. 4). In contrast, bright green fluorescence was detected on astrocytes throughout the 18 h incubation, a staining pattern very similar to that observed with MAGI cells exposed to fusion-incompetent 517B-GFP (middle and bottom panels in Fig. 4). Longer follow up studies revealed that HIV-GFP remains associated with HFA for up to 48 h after initial exposure without significant loss of the fluorescence signal (data not shown). Similar results were obtained in three independent experiments. These results indicate that HIV-1 binds efficiently and stably to the majority of cells in our HFA cultures and that most of the HFA-bound virus, unlike that bound to MAGI cells, fails to internalize. We cannot definitively localize the GFP-labeled virions by the microscopy method used, but by analogy to fusion-deficient 517B (Fig. 4), one possible reason for inefficient entry of HIV-1 into HFA is that virion binding is not followed by virus-cell fusion, at least for the bulk of virus visualized by our binding method. Under the conditions of these experiments, virion phagocytosis by HFA [78] was not detected but it cannot be excluded.

### HIV-1 expression occurs in a small subset of HFA

**Figure 5:**
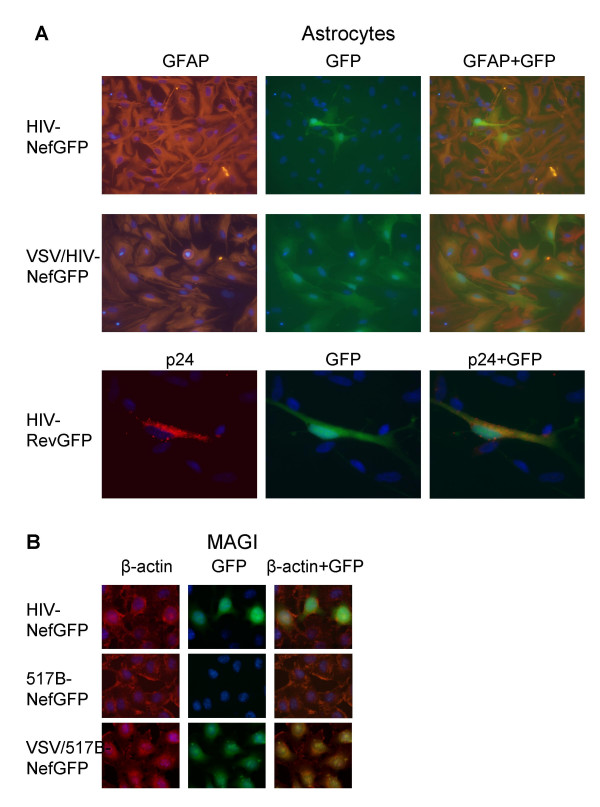
**Expression of HIV-1 regulatory and structural genes by infected HFA and control cells**. A) HFA were infected with VSV-G pseudotyped NL4-3 encoding a NefGFP fusion protein (first row), with native NL4-3 encoding NefGFP (second row) or encoding RevGFP (third row). Two days after infection HFA were stained using specific antibodies for GFAP (first and second rows) or p24 (third row) and with DAPI and Texas Red-labeled secondary antibodies. Images of red and green fluorescence were superimposed (third column). B) MAGI cells were infected with NL4-3 encoding NefGFP (first row), or fusion incompetent 517B encoding NefGFP (second row), or 517B-NefGFP pseudotyped by VSV-G (third row). Two days after infection cells were stained with anti-actin, DAPI, and Texas Red-labeled secondary antibody and images of red and green fluorescence were superimposed (third column).

Previous studies have shown that HIV-1-infected astrocytes express high levels of regulatory viral proteins such as Rev or Nef [27,63] indicating the utility of these protein markers of astrocyte infection. Therefore, to facilitate detection of HIV-1 expressing HFA, we constructed either NL4-3 or fusion-incompetent 517B with genes encoding fusion proteins of GFP with either Nef or Rev to visualize HIV-1 infections at the single cell level in HFA. An additional advantage of these constructs was that since these proteins are not carried in virions, their detection requires new viral protein synthesis by infected cells. HFA were infected by HIV-NefGFP or HIV-RevGFP or by VSV-G pseudotyped HIV-1 carrying NefGFP and infected cells were examined by fluorescence microscopy 48 h later (Fig. 5A). Consistent with our previous study [66], the bulk of HFA could be productively infected by VSV-G pseudotyped HIV-1 as shown in the detection of NefGFP. In contrast, only about 1% of HFA expressed NefGFP after infection by HIV-1 using its native envelope protein, gp120. To address the issue of the relative expression of structural versus regulatory genes, HFA were infected with virus encoding RevGFP and then were stained for expression of p24 (lower panel in Fig. 5A). All cells that expressed RevGFP also expressed p24 indicating no defect in Rev functions or structural gene expression. To validate this approach, a similar experiment was performed using CD4-positive MAGI cells and the fusion-mutant 517B (Fig. 5B). NefGFP was efficiently expressed after NL4-3 infection but not after 517B expression, indicating that Nef detection required virus entry and synthesis of viral protein. Expression of NefGFP by 517B could be rescued by pseudotyping with VSV-G (lower panel in Fig. 5B), in direct analogy to studies with HFA in Fig. 5A, and confirming that the 517B mutation in gp41 prevents the mutant virus internalization and expression [77]. Similar results were obtained in three independent experiments. These results support the interpretation that NefGFP expression is suitable for detection of *de novo *viral protein synthesis as described for HFA in Fig. 5A.

### Induction of cytokines by exposure of HFA to HIV-1

**Figure 6:**
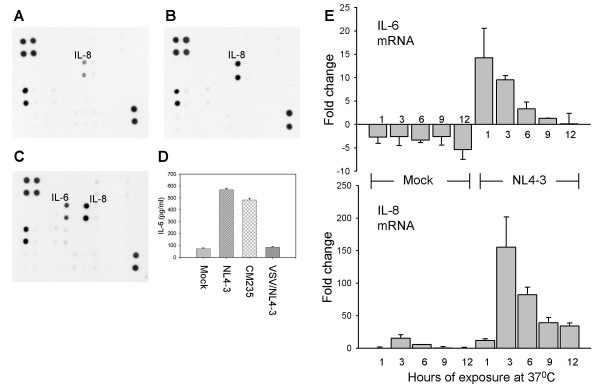
**Induction of cytokines by exposure of HFA to HIV-1**. A-C) HFA were mock-infected (panel A); infected with VSV/NL4-3, a VSV-G pseudotyped NL4-3 incapable of expressing gp120 (panel B), or infected with NL4-3 (panel C), washed, and after 24 h incubation cell supernatants were collected and applied to RayBio Human Cytokine Antibody Array 3.1. The map of antibodies present on the array can be accessed at . D) HFA were infected as indicated, washed, and after 24 h incubation supernatants were collected for assay of IL-6 levels by ELISA. E) The expression of IL-6 (upper panel) and IL-8 (lower panel) mRNA by HFA was measured by qPCR at the indicated times after mock infection or NL4-3 infection.

Previous studies have shown that viral envelope protein gp120 can induce a range of neuroinflammatory products in primary astrocytes in culture [46,79], but intact HIV-1 was tested of this activity only in transformed cell lines [43,80]. Here we tested whether HIV-1, consistent with its ability to efficiently bind to HFA, can induce expression of any of a panel of cytokines and chemokines detectable by multi-protein blots (Fig. 6A–C). Culture supernatants from HFA 24 h after mock infection, infection with VSV-G pseudotyped NL4-3 that carries an incapacitating mutation in gp120 (VSV/NL4-3), and infection with native HIV-1/NL4-3 all contained IL-8, but the chemokine expression was increased by VSV/NL4-3 and native HIV-1 exposure. In contrast, IL-6 was only detected after exposure of astrocytes to native HIV-1 (Fig. 6C). To investigate the virus specificity of the IL-6 response, we exposed HFA to X4 NL4-3; CM235, an R5 HIV-1; or VSV/NL4-3 and then tested the expression of IL-6 by ELISA (Fig. 6D). Both the X4 and the R5 HIV-1-induced IL-6 protein expression. However the replication competent VSV/NL4-3 (which lacks gp120) failed to induce IL-6 within the test time frame, strongly suggesting that gp120 binding and no other HIV-1 function was the initiator of the IL-6 response. To determine the kinetics of the observed responses to HIV-1, HFA were mock-infected or infected by NL4-3 and at time increments between 1 h and 12 h cells were harvested for quantitative real-time PCR assay of their levels of IL-6 and IL-8 transcripts (Fig. 6E). The RNA measurements were standardized by the levels of glyceraldehyde phosphate dehydrogenase RNA and data are expressed as fold change relative to cells harvested at 0 time, immediately prior to infection or mock infection (Fig. 6E). Both transcripts were induced rapidly after HIV-1 exposure and then declined by 9–12 h. In contrast in mock-infected cells IL-6 expression was slightly reduced in culture and IL-8 expression increased 15-fold after 3 h in culture, the latter consistent with the results of multi-protein blots (Fig. 6A–C). Similar results were obtained in at least seven independent experiments utilizing different preparations of astrocytes and different virus batches. These results indicate that the near-universal binding of HIV-1 to HFA results in a rapid induction of expression of two arms of innate immunity, interferon-based and chemokine-based.

## Discussion

The results presented here reveal a new basis for functional responses of astrocytes to HIV-1. We show that effectively all of the cells in cultured fetal astrocyte populations bind HIV-1 but only a small minority of cells permits HIV-1 infection. HIV-1 binding is sufficient to trigger rapid synthesis of IL-6, one of the major innate immune effectors associated with HIV-1 neuropathogenesis [13] and it increases the expression of IL-8, a potent chemokine [81]. These results suggest that HIV-1 affects astrocyte physiology on a cell population-wide basis primarily through binding to cells and binding-induced signaling, rather than infection.

Resolution at the single cell level permitted us to draw these conclusions. We employed a GFP-Vpr tagged "green HIV-1" [72] and infectious HIV-1 expressing Rev- or NefGFP fusion proteins to assess the relative efficiency of virus binding, entry, and expression by fluorescence microscopy. Binding was clearly the most pronounced step of HIV-1 interaction with astrocytes. HIV-1 bound to the majority of astrocytes in our cell cultures, similar to its interaction with CD4-positive cells as visualized by microscopy and exceeding binding to some CD4-positive cells as measured by p24 association (Figs. 1 and 3). The binding observed was specific as indicated in its competition with excess unlabeled virus (Fig. 1) and the absence of binding to unrelated CD4-negative cells (Figs. 2 and 3). The HIV-1 interaction with astrocytes is likely to be mediated by the viral envelope glycoprotein gp120 because preincubation of GFP-Vpr tagged virions with soluble CD4 prevented HIV-1 binding in our study (Fig. 1) and it was shown to block astrocyte infection by HIV-1 in a previous report [61]. Furthering supporting this conclusion, another study demonstrated that recombinant gp120 binds to a single-class binding site on human fetal astrocytes and that the affinity of this interaction approaches that of the gp120-CD4 binding [60]. Taken together, our results directly show that, like human T lymphocytes and macrophages, human astrocytes can be classified as a cell type fully competent to bind HIV-1. We do not know at present whether adult astrocytes exhibit similar HIV-1 binding characteristics as fetal cells used in the present work, but it should be noted that fetal astrocytes are an acceptable model for study of many receptor-ligand interactions including those with neurotransmitters, cytokines, and growth factors [57,82,83]. Given the size of the astroglial compartment [17], our findings raise the possibility that astrocytes present a major target for absorbing HIV-1 and gp120 produced in HIV-1-infected brain.

The cell surface receptor(s) responsible for HIV-1 binding to astrocytes has not been conclusively identified although several candidate proteins have been described [78,84]. CD4 serves as the binding receptor for HIV-1 on T lymphocytes and macrophages and binding to CD4 facilitates binding to co-receptors required for virus-cell fusion [85]. Astrocytes do not express CD4 [60] but have been shown to variously display viral co-receptors including CCR3, CCR5, and CXCR4 [74,75] and in some other systems the viral co-receptor is sufficient to bind immunodeficiency viruses. CD4-independent HIV-2 and SIV have shown to bind and enter T cells through CXCR4 [86,87] and the major receptor for some FIV on T cells appears to be CXCR4 [88]. Nevertheless, monoclonal antibodies that bind CXCR4, CCR3, or CCR5 did not block HIV-1 binding to HFA. Indeed anti-CXCR4 treatment slightly enhanced HIV-1 binding. A more recent study identified the chemokine receptor D6 as a receptor on HFA for certain HIV-1 and HIV-2 clones, but NL4-3 that we employed here could not enter HFA via D6 [84]. These results rule out these chemokine receptors as the major receptors for HIV-1/NL4-3 on HFA but leave open the identity of the receptor.

Compared to T lymphocytes and macrophages, astrocytes undergo only limited infection with HIV-1 *in vitro *and *in vivo *[7,27,28,63,64]. Explanations advanced by our laboratory and others include inefficient virus entry [65-67] and intracellular restrictions to HIV-1 expression [63,89]. This work further clarifies the relative contributions of these mechanisms. By analysis at the single cell level we show that the majority of astrocytes in our cell populations are refractory to HIV-1 infection despite universal binding of virus to HFA (Figs. 1 and 5). We attribute this disparity to inefficient virus entry into the majority of astrocytes that bind HIV-1. The block at entry was indicated by i) different dynamics of HIV-GFP association with HFA and CD4-positive cells at 37°C as visualized by fluorescence imaging; ii) the similarity between GFP-Vpr tagged NL4-3 and entry-defective 517B viruses in their respective target cell associations by the same method; and iii) the demonstration at a single cell level that circumventing restricted HIV-1 entry by use of VSV-G pseudotyped virus permits efficient infection of HFA, as also shown by cell population analyses in our previous studies [33,34,66] and consistent with other reports [34,67,84,90]. The 517B construct of HIV-1 contains a mutation in the fusion domain of gp41 that incapacitates virus-cell fusion and virus entry [77]. Based on analogy with 517B revealed here, HIV-1 binding to HFA may not generally be followed by virus-cell fusion, resulting in inefficient entry in most of the cells exposed to the virus. The mechanism of HIV-1 entry into the minority of HFA that permit virus infection is also uncertain at present, although at least one study suggested it is by endocytosis mediated by a mannose receptor [78].

Although GFP-Vpr tagged HIV-1 is a useful tool to investigate initial virus-cell interactions it is not suitable for enumerating the fraction of astrocyte that does permit virus infection because it does not encode GFP. We used for that purpose infectious HIV-1 expressing RevGFP and NefGFP fusion proteins. Other investigators have also employed RevGFP constructs to investigate its function on astrocytes outside HIV-1 infection [91]. RevGFP and NefGFP are not subject to the proposed intracellular restrictions to HIV-1 replication in astrocytes [63,89] and thus their detection accurately marks all HFA in our cell populations that allow virus entry and expression. We found that Rev and Nef were expressed only in a small fraction of cells, about 1% (Fig. 5), consistent with previous reports [68,69]. Of note, the HIV-RevGFP-infected astrocytes also expressed p24 in double-label analysis (Fig. 5). This indicates that some astrocytes may permit completion of the HIV-1 replicative cycle, at least during acute infection and consistent with our studies with VSV-G/HIV and some other reports [33,66,67,84,90]. However, our studies do not exclude the possibility that following transition to chronic infection astrocytes may impose some restrictions on HIV-1 expression, as indicated by establishment of inducible HIV-1 latency in chronically infected HFA *in vitro *[92] and limited HIV-1 expression by infected cells *in vivo *[7]. Regardless of the mechanism of long-term HIV-1 infection in astrocytes, our work makes clear that only a small fraction of HFA competent to bind HIV-1 is susceptible to HIV-1 entry and expression at any given time. It is unknown at present whether HIV-1 infects cells randomly or it targets some yet undefined astrocyte subpopulation [18,19]. More research is needed to identify HIV-1 susceptible cells in order to understand the pathogenic potential of this infection.

In addition, binding of HIV-1 through gp120 has functional consequences for HFA as previously reported [54,55] and as shown here. Our results demonstrate that HFA can be triggered to IL-6 expression by HIV-1 binding but that HIV-1 infection by VSV-G/pseudotyped, gp120-negative virus capable of expressing all other viral genes cannot trigger this gene expression. These findings indicate that gp120 binding and no other viral function activated IL-6 expression, at least in this 24 h exposure period. Indeed, the induction of the IL-6 transcript was maximal at the first time point tested, 1 h after virus exposure. IL-8 induction peaked somewhat later, at 3 h after exposure. Previous studies have shown that HIV-1 Tat can activate astrocytes to IL-8 production, although the activation was about 10-fold less than that observed here [44]. Because we find a small minority of cells susceptible to HIV-1 infection, it is more likely that the IL-8 expression observed here resulted from gp120 binding to HFA than from virus replication and Tat production. IL-8 is a major chemotactic factor for a variety of leukocytes [81] and increased IL-6 in the central nervous system has been associated with brain disease in SIV-infected macaques and has been implicated as a critical controlling element in brain disease in the SIV-macaque model [13,93]. Our observation raises the possibility that exposure of astrocytes in the brain to HIV-1 or to gp120 is responsible for the observed increase in this key antiviral cytokine, independently of virus replication in the brain. If the results reported here faithfully reproduce the behavior of astrocytes in the brain, then this numerous and essential brain cell type is exquisitely responsive to HIV-1 and its responses may be a major factor in HIV-1 neuropathogenesis.

## Conclusion

Our results indicate that HIV-1 binding to human astrocytes, although extensive, is not generally followed by virus entry and replication. Astrocytes respond to HIV-1 binding by rapidly increased cytokine production suggesting a role of this virus-brain cell interaction in HIV-1 neuropathogenesis.

## Methods

### Plasmid construction

pEGFP-Vpr plasmid was obtained from Dr. T. Hope (Northwestern University). To generate the vector encoding infectious NL4-3 expressing NefGFP, the wild-type HIV-1 pNL4-3 vector (from NIH AIDS Research and Reference Reagent Program, Germantown, MD) was PCR amplified using the *Pfu *DNA polymerase (Stratagene, La Jolla, CA) with primer pair 5'-CGATTAGTGAACGGATCCTTAG-3' and 5'-CTTGCTCACCATAGCGGCCGCCCACTTGCCACCCATCTTATA-3'; pGFP-C1 was PCR amplified with primer pair 5'-ATGGGTGGCAAGTGGGCGGCCGCTATGGTGAGCAAGGGCGAG-3' and 5'-GACGTCGCTCGAGTTACTTGTACAGCTCGTCC-3'. The PCR products were run and separated on 1% agarose gel, purified by Ultrafree-DA tubes (Millipore, Billerica, MA), then used as template for PCR with primer pair 5'-CGATTAGTGAACGGATCCTTAG-3' and 5'-GACGTCGCTCGAGTTACTTGTACAGCTCGTCC-3'. The second round PCR product which joined above two fragments (HIV-1 and GFP coding sequence) was ligated to *Bam*H I and *Xho *I sites of pNL4-3 plasmid. To generate vector HIV/RevGFP, HIV-1/Lai (from NIH AIDS Research and Reference Reagent Program) was PCR amplified using the Pfu DNA polymerase with primer pair 5'-CGATTAGTGAACGGATCCTTAG-3' and 5'-CTCGCCCTTGCTCACCATTTCTTTAGTTCCTGACTCCAA-3'; pGFP-C1 was PCR amplified with primer pair 5'-TCAGGAACTAAAGAA*ATG*GTGAGCAAGGGCGAGGAGCTG-3' and 5'-GACGTCGCTCGAGTTACTTGTACAGCTCGTCC-3'. The PCR products were run and separated on 1% agarose gel, purified by Ultrafree-DA tubes (Millipore), then used as template for PCR with primer pair 5'-CGATTAGTGAACGGATCCTTAG-3' and 5'-GACGTCGCTCGAGTTACTTGTACAGCTCGTCC-3'. The second round PCR product which jointed above two fragments was ligated to *Bam*H I and *Xho *I sites of pHIV-1/Lai plasmid. The construction of all of the above plasmids were confirmed by restriction enzyme digestion or sequencing. The NL4-3-517B was constructed by excising the envelope coding sequence from pHXB2-517B [77], obtained from Dr. J. Sodroski, Harvard Medical School, and inserting it into the *Sal *I and *Bam*H I sites of pNL4-3. Plasmid encoding infectious CM235 [94] was kindly provided by Dr. P. Ehrenberg (Walter Reed Army Institute of Research, Rockville, MD).

### Cell culture, transfection and infection

HFA were isolated from second trimester human fetal brains (gestational age 16–19 weeks) obtained from elective abortions in full compliance with National Institutes of Health guidelines. Highly enriched HFA preparations were obtained by high-density culture in the absence of growth factors in F12 DMEM (Invitrogen, Carlsbard, CA) containing 10% fetal bovine serum (FBS) and antibiotics essentially as described [66]. The cells were propagated in the same medium at 2–5 × 10^4 ^cells/cm^2 ^and subcultured weekly up to 4 times. Experiments were performed on HFA in passage 3; at this stage >99% of cells stained positive for the astrocyte marker GFAP and no microglial contamination was detectable by anti-CD68 staining (not shown; [33,66]). 293T, HeLa and HeLa-CD4-LTR-βGal (MAGI) cells were obtained from the NIH AIDS Research and Reference Reagent Program; the cells were cultured in DMEM supplemented with 10% FBS and penicillin-streptomycin (100 U/ml). Transfections were performed by the standard calcium phosphate precipitation method which usually in our hands has over 50% transfection efficiency in 293T cells. PBL from healthy donors were activated for 48 h with phytohemagglutinin (PHA) and were then cultivated in RPMI 1640 medium supplemented with 10% FBS. CEM cells were cultured in RPMI-1640 containing 10% FBS.

### Concentration and purification of viral particles

To prepare HIV-GFP 293T cells were cotransfected with an EGFP-Vpr and a designated HIV-1 plasmid and after two days supernatant was collected and virus content determined using the HIV-1 Ag ELISA kit, Coulter, Hialeah, FL. Alternatively, PBL were infected with different strains of HIV-1, after 5 days, supernatant was collected, filtered through 0.22-μm-pore-size filters, layered over a 20% sucrose cushion in PBS, and centrifuged for 1 h at 20,000 × *g*. The viral pellet was washed once, resuspended in PBS, and virus content was determined by p24 ELISA.

### Determination of virus binding by measurement of cell-associated p24

HFA, CD4-negative 293T or HeLa, and CD4-positive-MAGI cells (2 × 10^5 ^cells/well in 12-well plates) were incubated with indicated virus preparations at a dose of 1 pg p24 per cell for 2 h at 4°C. This dose is equivalent to one infectious unit (I.U.) per cell as determined by virus infectivity assay in MAGI cells [73]. To evaluate inhibition of virus binding, cells were exposed to human anti-CXCR4 (12G5), anti-CCR5 (5C7) or anti-CCR3 (7B11) monoclonal antibodies (NIH AIDS Research and Reference Reagent Program) at 1 μg/ml for 30 min prior to virus exposure at either 37°C or 4°C in a final volume of 500 μl and then exposed to virus for 1 h at 4°C. At the end of the incubation period, cells were vigorously washed five times with PBS to remove unabsorbed virus and lysed with 300 μl of PBS containing 0.5% NP40. The lysates were cleared by centrifugation (10,000 × *g *for 5 min) and the p24 content of the lysate was determined by ELISA. The percentage of virus particles bound by cells was calculated by the following formula: [(pg p24 associated with cells)/(pg input p24) × 100]. All virus binding or infection assays were performed in duplicate or triplicate.

### Determination of GFP-Vpr virus binding and entry by fluorescence microscopy and image analysis

For binding experiments, HFA, HeLa and MAGI cells were grown in 12-well plates with round coverslips. Cells were exposed to HIV-GFP virions at 1 pg viral p24 per cell for 1 h at 4°C. To compete for HIV-GFP binding, cells were exposed to 10-fold excess of unlabeled NL4-3 virions for 1 h at 4°C prior to exposure to HIV-GFP virions. To inhibit binding, HIV-GFP virions were pretreated with 20 μg/ml of soluble CD4 prior to incubation with cells. Virus-cell mixtures were then washed five times with PBS to remove unabsorbed virus; washed cells were fixed with 2% paraformaldehyde in PBS, stained with 1 ng/ml of DAPI in methanol for detection of nuclei, stained with primary antibodies, washed, stained with Texas Red linked cognate secondary antibodies (Amersham, Piscataway, NJ), washed, air dried, and mounted in glass slides with 50% glycerol in PBS. To follow virus internalization, cells were exposed to HIV-GFP virions at 4°C for 1 h, washed, supplemented with appropriate media, cultured 37°C for the designated periods of time, and fixed and observed or stained as described above. Fluorescence images were captured by using a digital video imaging microscope system consisting of a Zeiss Model Axioplan 2 microscope (Carl Zeiss, Thornwood, NY) with a HAMAMATSU ORCA-ER digital camera (HAMAMATSU Corp, Bridgewater, NJ) and Openlab software (Improvision, Lexington, MA).

### Human cytokine array

HFA were mock infected or incubated with designated virus for 5 h at 37°C, cells were washed five times and cultured in low serum medium overnight. Supernatant were collected and incubated with the Human Cytokine Array 3.1 multiprotein blot (Ray Biotech, Norcross, GA) membrane for 2 h at room temperature. After decanting the samples from container, the membrane was washed three times. Biotinylated anti-human cytokine antibodies were added and incubated at room temperature for 2 h. This was followed by washing, incubation with streptavidin conjugated peroxidase, and visualization by enhanced chemiluminescence system (Amersham).

### Quantitative real-time RT-PCR (qPCR)

HFA were mock-infected or infected with 1 pg p24 per cell of NL4-3, and RNA was extracted and cDNA was synthesized as described above. qPCR was conducted as described [95] using Taqman chemistry with human IL-6, IL-8, and GAPDH probes and primers designed using Primer Express v.1.0 (Applied Biosystems). PCR reaction contained 5 μl of ABI 2× Universal Master Mix, 1.25 μl of each forward and reverse primers at 200–900 nM, 1 μl of probe at 50–200 nM, and RNAase/DNAase free water. All reactions were performed in duplicate and were run in an ABI 7500. Raw data were analyzed using the Sequence Detection Software (ABI); relative quantitation employed the comparative threshold cycle method (Applied Biosystems Technical Bulletin #2). Signals were normalized to signals of the levels of GAPDH transcripts and are presented as fold changes versus 0 time, with cells collected before infection or mock infection.

### IL-6 ELISA

The IL-6 protein level in cell growth medium was detected by human IL-6 ELISA development system (R&D Systems Inc, Minneapolis, MN). Briefly, 96-well plates were coated with anti-human IL-6 monoclonal antibodies overnight, samples were added to the designated wells with 100 μl per well and incubated at RT for 2 h. Fluid was aspirated and the wells were washed three times with washing buffer. Then 100 μl biotinylated anti-human IL-6 was added to each well and incubated at RT for 2 h. This was followed by washing, incubation with streptavidin conjugated peroxidase and color development.

### Antibodies and other reagents

Anti-capsid (HIV-1 p24) monoclonal antibody was obtained from the NIH AIDS Research and Reference Reagent Program. Anti-β-actin monoclonal antibody was purchased from Sigma (St. Louis, MO). Anti-GFAP polyclonal antibodies were purchased from DAKO Corporation (Carpinteria, CA).

## Abbreviations

HIV-1: human immunodeficiency virus type 1: HFA: human fetal astrocytes; HAD: HIV-associated dementia; GFP: green fluorescence protein

## Authors' contributions

Jinliang Li designed experiments, conducted most of the work, and wrote draft of the manuscript; Galina Bentsman established, maintained, and characterized cultures of human fetal astrocytes and conducted some of the analytical assays; Mary Jane Potash conducted critical data analysis and interpretation and co-wrote the manuscript; David Volsky planned, designed, and supervised the work, analyzed data, and co-wrote the manuscript.
